# Active disturbance rejection control based on soft computing techniques for electric power steering to improve system performance

**DOI:** 10.1371/journal.pone.0324600

**Published:** 2025-06-06

**Authors:** Tuan Anh Nguyen

**Affiliations:** Division of Automotive Engineering, Thuyloi University, Hanoi, Vietnam; Federal University of Technology - Parana, BRAZIL

## Abstract

Electric Power Steering (EPS) systems enhance driving comfort and safety. However, their performance often degrades under varying operating conditions due to external disturbances and modeling uncertainties. Traditional control methods, which typically rely on fixed parameters or neglect disturbance dynamics, struggle to maintain robustness and adaptability across diverse scenarios. This article presents an improved control strategy integrating Active Disturbance Rejection Control (ADRC) with advanced soft computing techniques to address these challenges. The proposed method introduces two key innovations: optimizing the tracking differentiator’s speed factor using a genetic algorithm and dynamically tuning state feedback control parameters through a fuzzy inference system. This hybrid approach enhances the disturbance rejection capability of ADRC and significantly improves system adaptability and tracking accuracy. Simulation results validate the effectiveness of the proposed controller, demonstrating low tracking errors (1.875% at low speed and 1.373% at high speed) and disturbance estimation accuracy exceeding 90%. Compared to conventional controllers, the proposed method exhibits superior robustness, reduced steady-state error, and improved performance across a wide range of operating conditions. These results confirm the potential of integrating ADRC with intelligent optimization techniques for advanced control in automotive mechatronic systems.

## 1. Introduction

Today, most cars have power-assisted steering (PAS) systems to lessen steering effort, enhance steering feel, and increase safety. The PAS systems are divided into three main types: Electric Power Steering (EPS), Hydraulic Power Steering (HPS), and Electrohydraulic Power Steering (EHPS) [1]. Baharom et al. confirmed that EPS systems outperformed conventional HPS [2]. In [3], Ramasamy revealed that energy efficiency was improved by about 3% when replacing HPS systems with modern EPS. In addition, EPS systems also had a positive impact on improving vehicle comfort and safety. An investigation conducted by Park et al. showed that using hybrid EPS systems could improve vehicle energy efficiency [4]. In addition, EPS systems have many other advantages, such as compact size, fast response speed, and environmental friendliness. Therefore, they are increasingly widely used to replace conventional mechanical steering systems.

### 1.1. EPS control review

Several studies on EPS control have been published recently. In [5], Guan et al. designed a Proportional-Integral-Derivative (PID) controller to calculate the desired pinion angle. The structure of this controller was quite simple. They used the MATLAB toolbox to find the PID parameters. However, this method did not bring high efficiency to the system. Cao and Zheng designed a fuzzy universe technique to tune the parameters for the PID controller [6]. To accomplish this, they only used trapezoidal membership functions. In [7], Hanifah et al. proposed two swarm intelligent methods called Particle Swarm Optimization (PSO) and Ant Colony Optimization (ACO) to find the optimal parameters for the PID controller. The ideal parameters were found based on minimizing the objective function. The simulation results in [7] revealed that the maximum assisted current was reduced from 25.02 A (conventional PID) to 24.98 A (PID-ACO) and 24.99 A (PID-PSO). Jung proposed a hardware-in-the-loop simulator for EPS control based on the PID method, which considered the crosswind effect [8]. Jung and Kim designed an adaptive disturbance observer to estimate the crosswind effect, considered a significant lateral disturbance [9]. Zheng and Wei proposed a phase-compensated fuzzy PI technique [10]. The parameters of the PI controller were adjusted dynamically by the fuzzy algorithm, which was formulated based on triangular and Gaussian membership functions. The algorithm designed in [10] performed better than conventional PI and traditional ADRC, although overshoot and signal delay still existed. A self-tuning technique for PID parameters called Back Propagation Neural Network (BPNN) was introduced in [11] by Li et al. This algorithm’s performance was dependent on the speed of the vehicle. The assisted torque generated by an electric motor was only sufficient for small and medium-sized vehicles. Therefore, Fu et al. designed dual-power EPS control to generate sufficient assisted torque for large-sized vehicles, such as buses, trucks, and others [12]. Although the PID algorithm has many advantages [13], such as low cost, simple design, high robustness, and systematicity, some limitations still exist. In [14], Zhao and Zhang confirmed that the steering wheel torque signal was overshoot when the conventional PID algorithm controlled the EPS system. In general, PID control is only suitable for simple linear systems characterized by Single Input and Single Output (SISO) [15]. In some cases, chattering and overshoot phenomena still occur when applying this technique to control the system, causing a deterioration in control performance [14,16,17].

To control Multiple Inputs and Multiple Outputs (MIMO) systems more efficiently, Chitu et al. designed a Linear Quadratic Regulator (LQR) technique to replace the conventional PID [18]. This method is based on minimizing the cost function [19]. In [20], Liu et al. utilized the Genetic Algorithm (GA) to optimize the parameters of LQR based on fault-tolerant control. Irmer and Henrichfreise designed a Linear Quadratic Gaussian (LQG) compensator to improve the performance of EPS control [21]. This method was based on the combination of LQR and LQE [22]. In [23], Yamamoto et al. designed a state feedback Linear Parameter-Varying (LPV) control law based on an H_∞_/H_2_ PI observer to control the performance of the EPS system. Experimental results showed that the observed error was significant. Capturing all system outputs, which serve as control inputs, is necessary for LQR control. Direct measurement of all outputs using physical sensors is costly and increases the influence of sensor noise, leading to increased steady-state errors [24].

Several robust control techniques have been applied to EPS systems to improve the controller performance. In [25], Kim et al. designed a Sliding Mode Control (SMC) technique with a disturbance observer for steering wheel torque tracking. An actual vehicle test was performed to verify the controller’s performance. However, the algorithm’s structure was not fully described, and various cases need further validation of the results. A robust nonlinear SMC controller based on the Extended State Observer (ESO) was proposed in [26] by Kim et al. The computational results in [21] revealed that the received signal was chattering due to disturbances. An application of SMC based on disturbance rejection for steering angle control was presented in [27] by Khasawneh and Das. The simulation results showed that the output signals were strongly chattering. Additionally, phase lag occurred, resulting in a degradation of control quality. An adaptive SMC technique for target torque tracking was proposed in [25] by Lee et al. The stability of the system was evaluated using the Lyapunov criterion. Although the steering wheel angle error was insignificant, the steering wheel speed and acceleration were significantly affected by the chattering phenomenon. Lee et al. proposed a new control method for automotive EPS systems [28]. This controller’s structure consisted of two modules that interacted with each other. Simulation results showed that the system error was quite large. A feedforward control mechanism for suppressing torque oscillation was proposed by Fu et al. [29]. Experimental results revealed that the raw data for signals were strongly chattering. A control method for mechatronic systems to eliminate the influence of external factors, called Active Disturbance Rejection Control (ADRC), was proposed in [30] by Fareh et al. According to Xiang et al., the structure of ADRC consists of an Extended State Observer (ESO), a Tracking Differentiator (TD), and the Linear State Error Feedback (LSEF) rule [31]. Ma et al. applied this technique to control the automotive EPS system. However, the performance improvement was not significant [32]. In [33], Na et al. designed an ADRC mechanism for steering wheel torque tracking. Although the system error was small, the chattering phenomenon remained strong for the steering angular speed. According to Zheng and Wei, the ADRC algorithm performed better than fuzzy PID and conventional PID under extreme working conditions [34]. Some nonlinear integrated control methods for EPS systems are highly complex [35,36], while intelligent computational techniques depend on the researcher’s experience [37–39]. Several applications of deep learning [40], machine learning [41], deep feature fusion [42], and parallel neural networks [43] can be highly effective in system control. However, the structures of these algorithms are relatively complex, and they depend heavily on the designer’s viewpoints [44]. In addition, the chattering effect still exists and causes a deterioration in control performance, leading to increased tracking errors [45].

Recently, numerous state-of-the-art control strategies for electrical devices have been proposed. In [46], Sun and You introduced machine learning and data-driven approaches for control applications. Furthermore, Sun et al. [47] developed a disturbance rejection control mechanism based on a first-order plus time-delay model.

Several high-performance and robust control methods have been developed in previous studies. In [48], Rsetam et al. presented the design of a cascaded ESO-based SMC. A combination of SMC and ADRC was introduced by Rsetam et al. in [49]. The application of a PI observer was discussed in [50]. In addition, various other control approaches for automotive steering systems were introduced in [51,52]. Overall, these methods have demonstrated impressive performance in controlling systems. However, their mathematical structures are complex.

### 1.2. Research gaps and motivation

Previous studies have demonstrated a certain degree of effectiveness in controlling automotive EPS systems. However, several critical limitations remain and warrant further investigation. Firstly, overshoot and phase delay persist when applying PID controllers, resulting in notable tracking errors. Secondly, the presence of sensor noise (from the measurement of multiple signals) significantly degrades the performance of LQR-based control strategies. Thirdly, control chattering is commonly observed when robust control methods such as SMC are implemented. In addition, external disturbances continue to adversely affect system performance, while the high computational complexity of some control algorithms poses challenges for practical, real-time implementation.

These unresolved issues highlight apparent research gaps. There is a pressing need to develop an integrated control strategy capable of attenuating external disturbances while maintaining system stability and precision. Moreover, optimizing control parameters is essential to ensure adaptability under varying operating conditions.

Although Active Disturbance Rejection Control (ADRC) has shown promising results in automotive mechatronic applications, employing a single ADRC configuration with fixed parameters fails to deliver consistent performance across diverse operational scenarios.

The above issues are considered as existing limitations. Addressing these challenges serves as the primary motivation for this article.

### 1.3. Key contribution

Motivated by the limitations identified in existing approaches, this article proposes an enhanced Active Disturbance Rejection Control (ADRC) framework designed to suppress external disturbances and improve system performance more effectively. Unlike conventional ADRC methods that rely on fixed parameter selection, the proposed approach employs a hybrid optimization strategy that combines a genetic algorithm with a dynamic fuzzy computing technique to tune the control parameters adaptively. This integrated methodology leverages the complementary strengths of both algorithms to reduce steady-state errors, suppress chattering, and mitigate phase lag, thereby enhancing control robustness and adaptability under varying operating conditions. The controller architecture remains simple and computationally efficient, facilitating real-time implementation in experimental settings. This novel combination and optimization strategy represent the primary contribution of this work, offering a distinct advancement over previous studies.

The article is organized into four sections. The introduction section presents an EPS control literature review and highlights the novel contributions. The second section details the mathematical model of the system, while the following section discusses the simulation results. Finally, the conclusion section mentions the proposed algorithm and outlines potential directions for future research.

## 2. Mathematical models

This section introduces the mathematical model of the EPS system and the proposed control algorithm.

### 2.1. EPS model

**Fig 1:**
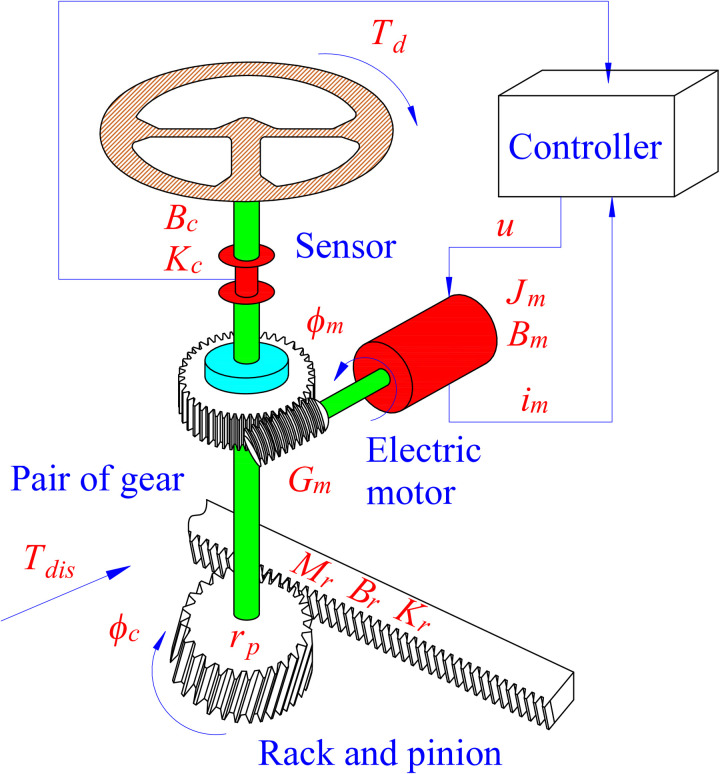
Electric power steering system.

A basic EPS system consists of a steering wheel, a steering column, a pinion and gear, an assisted motor, a pair of motor gears, sensors, and an ECM (Fig 1). The operating principle of the steering system is described by Equations 1 and 2, which are established based on the D’Alembert principle. The effects of torsional stiffness and friction are considered in these equations. The dynamics of the actuator are illustrated by Equation 3. The equivalent quantities are mentioned in (4) and (5).

The symbols used in the above equations are listed in Table 1. The system specifications are referenced from CARSIM software and several other articles [53,54].

**Table 1:** Technical symbols.

Symbol	Meaning	Unit	Value
*ϕ*_*c*_	Steering column angle	rad	–
*ϕ*_*m*_	Steering motor angle	rad	–
*B*_*c*_	Steering column damping	Nms/rad	0.063
*B*_*eq*_	Equivalent damping	Nms/rad	–
*B*_*m*_	Steering motor damping	Nms/rad	0.0044
*B*_*r*_	Rack damping	Ns/m	3330
*G*_*m*_	Motor gear ratio	–	18.5
*i*_*m*_	Assisted current	A	–
*J*_*c*_	Inertia moment of the steering column	kgm^2^	0.062
*J*_*eq*_	Equivalent moment of inertia	kgm^2^	–
*J*_*m*_	Inertia moment of the steering motor	kgm^2^	0.0004
*K*_*c*_	Steering column stiffness	Nm/rad	130
*K*_*t*_	Steering motor torque coefficient	Nm/A	0.054
*L*_*m*_	Steering motor inductance	H	0.006
*M*_*r*_	Rack mass	kg	27
*R*_*m*_	Steering motor resistance	Ω	0.43
*r*_*p*_	Pinion radius	m	0.006
*T*_*d*_	Driver torque	Nm	–
*T*_*dis*_	Disturbances	Nm	–
*u*	Control signal	V	–


$$J_{c}\frac{d^{2}}{dt^{2}}\phi_{c}+B_{c}\frac{d}{dt}\phi_{c}+K_{c}\phi_{c}=\frac{K_{c}}{G_{m}}\phi_{m}+T_{d}$$ (1)

$$\frac{K_{c}}{G_{m}}\phi_{c}+K_{t}i_{m}-\frac{T_{dis}}{G_{m}}=J_{eq}\frac{d^{2}}{dt^{2}}\phi_{m}+B_{eq}\frac{d}{dt}\phi_{m}+\frac{K_{c}+K_{r}r_{p}^{2}}{G_{m}^{2}}\phi_{m}$$ (2)

$$K_{t}\frac{d}{dt}\phi_{m}+L_{m}\frac{d}{dt}i_{m}+R_{m}i_{m}=u$$ (3)

$$J_{eq}=J_{m}+\frac{r_{p}^{2}}{G_{m}^{2}}M_{r}$$ (4)

$$B_{eq}=B_{m}+\frac{r_{p}^{2}}{G_{m}^{2}}B_{r}$$ (5)

Disturbances (*T*_*dis*_) are determined by two components: external disturbances (*T*_*edis*_) and internal disturbances (*T*_*idis*_), according to (6). Internal disturbances are caused by the steering process, which depends on the lateral tire force, calculated according to (7), where *d*_*c*_ is caster trail, *d*_*n*_ is knuckle arm length, *λ*_*kin*_ is kingpin angle, *λ*_*cas*_ is caster angle, and *F*_*y*_ is lateral tire force. In contrast, external disturbances originate from the impact of the external environment, such as roughness on the road, weather, crosswinds, and others.


$$T_{dis}=T_{idis}+T_{edis}$$ (6)

$$T_{idis}\approx r_{p}d_{c}\frac{cos^{2}\left(\lambda_{kin}\right)cos^{2}\left(\lambda_{cas}\right)}{d_{n}}F_{y1}$$ (7)

**Fig 2:**
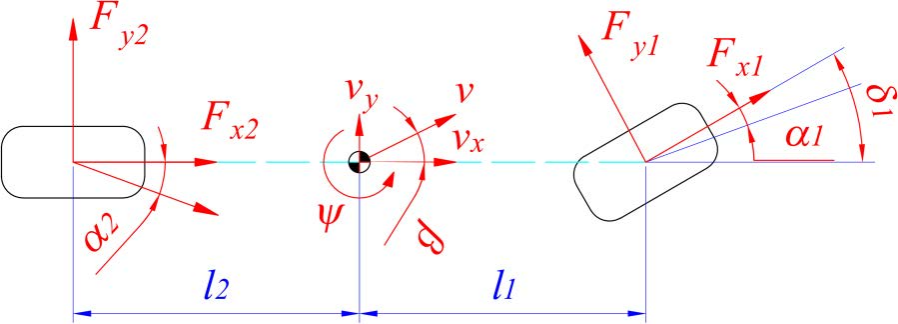
Vehicle dynamics model.


A linear dynamic model calculates the variation of lateral tire force (Fig 2). The tire is assumed to deform linearly, so *F*_*y*_ is determined by (8). The tire cornering stiffness (*C*_*α*_) is a known constant, while the tire slip angle (*α*) depends on the velocity (*v*), steering angle (*δ*), and yaw angle (*ψ*), described in (9). The variation of yaw angle with time is determined by Equations 10–12. Some previous studies assumed that *T*_*dis*_ was known in advance instead of being calculated from the dynamic model [55–57]. In contrast, Nguyen developed a spatial-dynamic model to calculate the total variation of *T*_*dis*_ [58,59]. However, the mathematical model was highly complex. In general, this calculation depends on the perspective of each author.

$$F_{yi}=-C_{\alpha i}\alpha_{i}$$ (8)

$$\begin{array}{c} \alpha_{i}=\frac{v_{y}+{\left(-1\right)}^{i-1}l_{i}\frac{d}{dt}\psi }{v_{x}}-\delta_{i} \end{array}$$ (9)

$$m\left(\frac{d}{dt}v_{x}-v_{y}\frac{d}{dt}\psi \right)=F_{x1}\text{cos}\delta +F_{x2}-F_{y1}\sin\delta$$ (10)

$$m\left(\frac{d}{dt}v_{y}+v_{x}\frac{d}{dt}\psi \right)=F_{y1}\text{cos}\delta +F_{y2}+F_{x1}\sin\delta$$ (11)

$$J_{\psi }\frac{d^{2}}{dt^{2}}\psi =l_{1}\left(F_{x1}\sin\delta +F_{y1}cos\delta \right)-l_{2}F_{y2}$$ (12)

The symbols mentioned in Equations 7 to 12 are listed in Table 2.

**Table 2:** Vehicle specifications.

Symbol	Meaning	Unit	Value
*λ*_*cas*_	Caster angle	deg	3
*λ*_*kin*_	Kingpin angle	deg	11
*C*_*α*_	Cornering stiffness	N/rad	40200
*d*_*c*_	Caster trail	m	0.03
*d*_*n*_	Knuckle arm length	m	0.3
*J*_*ψ*_	Yaw inertia moment	kgm^2^	3040
*l*_1_	Front axle distance	m	1.0
*l*_2_	Rear axle distance	m	1.5
*m*	Vehicle mass	kg	1580

The relationship between the lateral velocity (*v*_*y*_) and the longitudinal velocity (*v*_*x*_) is represented by Equations 13 and 14, where *β* is the heading angle.


$$v_{x}=v\cos\beta$$ (13)

$$v_{y}=v\sin\beta$$ (14)

Assume the steering angle is slight and the vehicle moves at a constant speed. The vehicle motion is written concisely as (15) and (16). By combining Equations 13 to 16, we can derive the matrix Equations 17. This matrix is used to determine the changes in heading angle and yaw angle.


$$m\left(\frac{d}{dt}v_{y}+v_{x}\frac{d}{dt}\psi \right)=\sum_{i=1}^{2}F_{yi}$$ (15)

$$J_{\psi }\frac{d^{2}}{dt^{2}}\psi =\sum_{i=1}^{2}{\left(-1\right)}^{i+1}l_{i}F_{yi}$$ (16)

$$\begin{array}{c} \left[\begin{array}{c} \frac{d}{dt}\beta \\ \frac{d^{2}}{dt^{2}}\psi \end{array}\right]=-\sum_{i=1}^{2}\left[\begin{array}{cc} \frac{C_{\alpha i}}{mv_{x}} & \frac{{\left(-1\right)}^{i+1}l_{i}C_{\alpha i}}{mv_{x}^{2}}+1\\ \frac{{\left(-1\right)}^{i+1}l_{i}C_{\alpha i}}{J_{\psi }} & \frac{l_{i}^{2}C_{\alpha i}}{J_{\psi }v_{x}} \end{array}\right]\left[\begin{array}{c} \beta \\ \frac{d}{dt}\psi \end{array}\right]+\left[\begin{array}{c} \frac{C_{\alpha 1}}{mv_{x}}\\ \frac{l_{1}C_{\alpha 1}}{J_{\psi }} \end{array}\right]\delta \end{array}$$ (17)

### 2.2. Control algorithm

Let the state variables be in order in Equation 18. Taking their derivatives, we get Equations 19 to 23.


$$\begin{array}{c} {\left[x_{i}\right]}^{T}={\left[\begin{array}{ccccc} \phi_{c} & \frac{d}{dt}\phi_{c} & \phi_{m} & \frac{d}{dt}\phi_{m} & i_{m} \end{array}\right]}^{T} \end{array}$$ (18)

$$\begin{array}{c} \frac{d}{dt}x_{1}=x_{2} \end{array}$$ (19)

$$\begin{array}{c} \frac{d}{dt}x_{2}=-\frac{K_{c}}{J_{c}}x_{1}-\frac{B_{c}}{J_{c}}x_{2}+\frac{K_{c}}{J_{c}G_{m}}x_{3}+\frac{T_{d}}{J_{c}} \end{array}$$ (20)

$$\begin{array}{c} \frac{d}{dt}x_{3}=x_{4} \end{array}$$ (21)

$$\begin{array}{c} \frac{d}{dt}x_{4}=\frac{K_{c}}{J_{eq}G_{m}}x_{1}-\frac{K_{c}+K_{r}r_{p}^{2}}{J_{eq}G_{m}^{2}}x_{3}-\frac{B_{eq}}{J_{eq}}x_{4}+\frac{K_{t}}{J_{eq}}x_{5}-\frac{T_{dis}}{J_{eq}G_{m}} \end{array}$$ (22)

$$\begin{array}{c} \frac{d}{dt}x_{5}=-\frac{K_{t}}{L_{m}}x_{4}-\frac{R_{m}}{L_{m}}x_{5}+\frac{1}{L_{m}}u \end{array}$$ (23)

This article proposes an innovative ADRC mechanism for controlling the EPS system. The algorithm consists of three fundamental components: the tracking differentiator, the extended state observer, and the state feedback control law. Compared with traditional control methods, the ADRC algorithm provides superior performance in eliminating the influence of disturbances under various operating conditions. In addition, the ADRC mechanism introduced in this article is improved by selecting optimal parameters based on the genetic algorithm and dynamic fuzzy computing technique to improve the controller performance and increase adaptability.

#### 2.2.1. ESO design.

It assumes only the sensor directly measures the first state variable (*x*_1_). As per Equation 24, the error between the measured and observed signals is represented as *e*_*θ*_. The remaining state variables are estimated using the ESO. Disturbances (*T*_*dis*_) are observed through an augmented variable (*x*_6_), as described in Equation 25. The structure of the ESO is demonstrated by Equations 26 to 31. The choice of the ESO structure is motivated by its capability to estimate and compensate for external disturbances. The mathematical model of the ESO is similar to the Luenberger observer. However, the most important difference lies in the augmented state variable, which estimates the disturbance. The observer gains (*θ*_*i*_) are chosen to ensure the stability of the observer.


$$\begin{array}{c} e_{\theta }=x_{1}-{\hat{x}}_{1} \end{array}$$ (24)

$$x_{6}=T_{dis}$$ (25)

$$\begin{array}{c} \frac{d}{dt}{\hat{x}}_{1}={\hat{x}}_{2}+\theta_{1}e_{\theta } \end{array}$$ (26)

$$\begin{array}{c} \frac{d}{dt}{\hat{x}}_{2}=-\frac{K_{c}}{J_{c}}{\hat{x}}_{1}-\frac{B_{c}}{J_{c}}{\hat{x}}_{2}+\frac{K_{c}}{J_{c}G_{m}}{\hat{x}}_{3}+\frac{T_{d}}{J_{c}}+\theta_{2}e_{\theta } \end{array}$$ (27)

$$\begin{array}{c} \frac{d}{dt}{\hat{x}}_{3}={\hat{x}}_{4}+\theta_{3}e_{\theta } \end{array}$$ (28)

$$\begin{array}{c} \frac{d}{dt}{\hat{x}}_{4}=\frac{K_{c}}{J_{eq}G_{m}}{\hat{x}}_{1}-\frac{K_{c}+K_{r}r_{p}^{2}}{J_{eq}G_{m}^{2}}{\hat{x}}_{3}-\frac{B_{eq}}{J_{eq}}{\hat{x}}_{4}+\frac{K_{t}}{J_{eq}}{\hat{x}}_{5}-\frac{1}{J_{eq}G_{m}}{\hat{x}}_{6}+\theta_{4}e_{\theta } \end{array}$$ (29)

$$\begin{array}{c} \frac{d}{dt}{\hat{x}}_{5}=-\frac{K_{t}}{L_{m}}{\hat{x}}_{4}-\frac{R_{m}}{L_{m}}{\hat{x}}_{5}+\frac{1}{L_{m}}u+\theta_{5}e_{\theta } \end{array}$$ (30)

$$\begin{array}{c} \frac{d}{dt}{\hat{x}}_{6}=\theta_{6}e_{\theta } \end{array}$$ (31)

The structure of TD is described in Equation 32, where *v*_0_ is the reference signal explained in Equation 33, and *k*_1_ and *k*_2_ are the model’s parameters, respectively. These parameters are chosen according to (34), with *r* being a positive constant, called speed factor. The selection of the speed factor in Equation 34 guarantees a double solution in the observer’s characteristic equation, thereby ensuring stability. At the same time, the output can quickly converge to the reference value with minor fluctuations. This selection method has been introduced and applied in [60].


$$\begin{array}{c} \left\{ \;\;\;\;\;\;\;\;\;\begin{array}{cc} & \frac{d}{dt}v_{1}=v_{2}\\ & \frac{d}{dt}v_{2}=-k_{1}\left(v_{1}-v_{0}\right)-k_{2}v_{2} \end{array}\right. \end{array}$$ (32)

$$v_{0}=x_{1ideal}$$ (33)

$$\begin{array}{c} \left\{ \;\;\;\;\;\;\;\;\begin{array}{cc} & k_{1}=r^{2}\\ & k_{2}=2r \end{array}\right. \end{array}$$ (34)

#### 2.2.2. Ideal assisted torque map.

**Fig 3:**
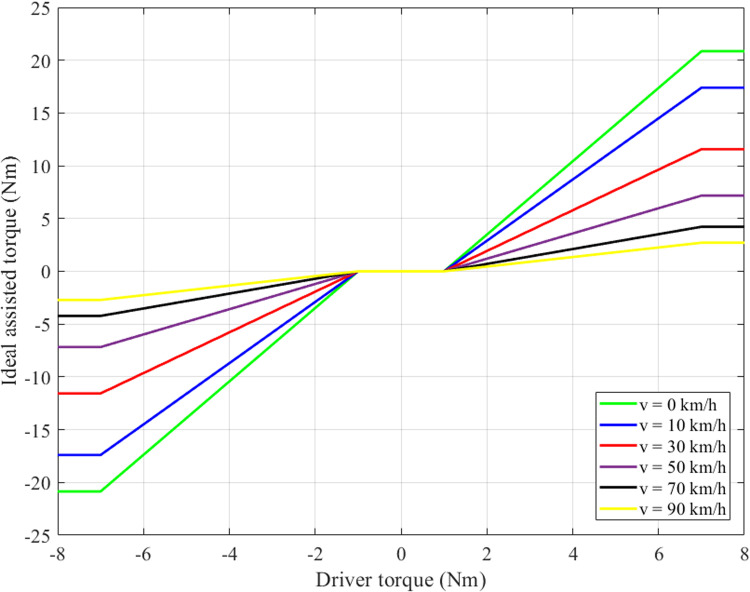
Ideal assisted map.

The reference signal (*x*_1*ideal*_) is taken from the ideal model, supported by the ideal assisted torque (*T*_*a_ideal*_). The value of the ideal assisted torque is looked up from the ideal assisted map (Fig 3). This map is constructed based on the saturated linear curves described by (35) and (36), where *a*_*i*_ are the coefficients of a quadratic polynomial.

A detailed analysis of Fig 3 indicates a linear relationship between the assisted torque and the driver torque. Moreover, the magnitude of the assisted torque is modulated by the vehicle’s speed: it decreases as the speed increases, and conversely. This adaptive control strategy enhances steering performance while preserving vehicle stability, particularly under challenging or hazardous driving conditions.


$$\begin{array}{c} T_{a\_ideal}=\left\{ \begin{array}{cc} 0 & T_{d}\leq T_{d\min}\\ f\left(v,T_{d}\right) & T_{d\min}<T_{d}<T_{d\max}\\ T_{a\max} & T_{d}\geq T_{d\max} \end{array}\right. \end{array}$$ (35)

$$f\left(v,T_{d}\right)=\left(T_{d}-T_{d\min}\mathrm{\backslash rightleft}(a_{1}v^{2}+a_{2}v+a_{3}\right)$$ (36)

#### 2.2.3. Control mechanism based on ADRC.

**Fig 4:**
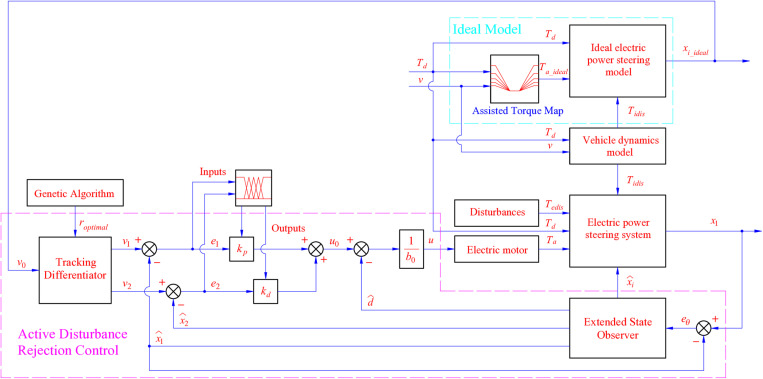
Proposed control scheme.

The control mechanism illustrated in Fig 4 comprises two main modules: the ideal and control models. The reference values (*x*_*ideal*_) are generated by an ideal model driven by the ideal assisted torque. The ADRC strategy is developed based on the ESO, with its parameters optimally tuned using soft computing techniques, including genetic and fuzzy algorithms.

The state feedback control law is introduced in (37), where *b*_0_ is the critical gain, and *u*_0_ is the initial control signal. The value of critical gain represents the uncertainty of the system. However, it is difficult to determine this precisely. This work simplifies the approach by assuming *b*_0_  = *b* = 1/*L*_*m*_. Total disturbances are determined according to (38).


$$\begin{array}{c} u=\frac{u_{0}-\hat{d}}{b_{0}} \end{array}$$ (37)

$$\begin{array}{c} \hat{d}=-\frac{K_{t}}{L_{m}}{\hat{x}}_{4}-\frac{R_{m}}{L_{m}}{\hat{x}}_{5}+\theta_{5}e_{\theta }+\left(\frac{1}{L_{m}}-b_{0}\right)u \end{array}$$ (38)

According to (39), a Proportional-Derivative (PD) controller determines the initial control signal. The system errors *e*_1_ and *e*_2_ are determined by (40) and (41), respectively, where *k*_*p*_ is a proportional parameter and *k*_*d*_ is a derivative parameter.


$$u_{0}=k_{p}e_{1}+k_{d}e_{2}$$ (39)

$$e_{1}=v_{1}-{\hat{x}}_{1}$$ (40)

$$e_{2}=v_{2}-{\hat{x}}_{2}$$ (41)

The proposed control mechanism in Fig 4 is shown in the form of block diagrams, which are explained in detail as follows:

- The ideal electric power steering model block is illustrated through Equations 1 to 5.
- The vehicle dynamics model block is established by Equations 6 to 17.
- The electric power steering system and ESO blocks are mentioned in Equations 18 to 34.
- The assisted torque map block is represented by Equations 35 and 36.
- The ADRC mechanism is presented in Equations 37 to 41.
- The GA block is illustrated by Equations 42–44.
- Finally, Equations 45 to 48 present the fuzzy inference system.

#### 2.2.4. Parameters tuning.

The parameters (*θ*_*i*_) mentioned in Equations 26 to 31 play an important role in maintaining the stability of ESO. In this work, we propose to use the pole placement method to find the appropriate parameters for ESO to ensure the stability and robustness of the system.

The choice of parameters *r*, *k*_*p*_, and *k*_*d*_ significantly influences the system’s performance. In this work, we propose to use the GA to find the optimal value of the speed factor (*r*), while *k*_*p*_ and *k*_*d*_ are determined by a dynamic fuzzy technique.

**Fig 5:**
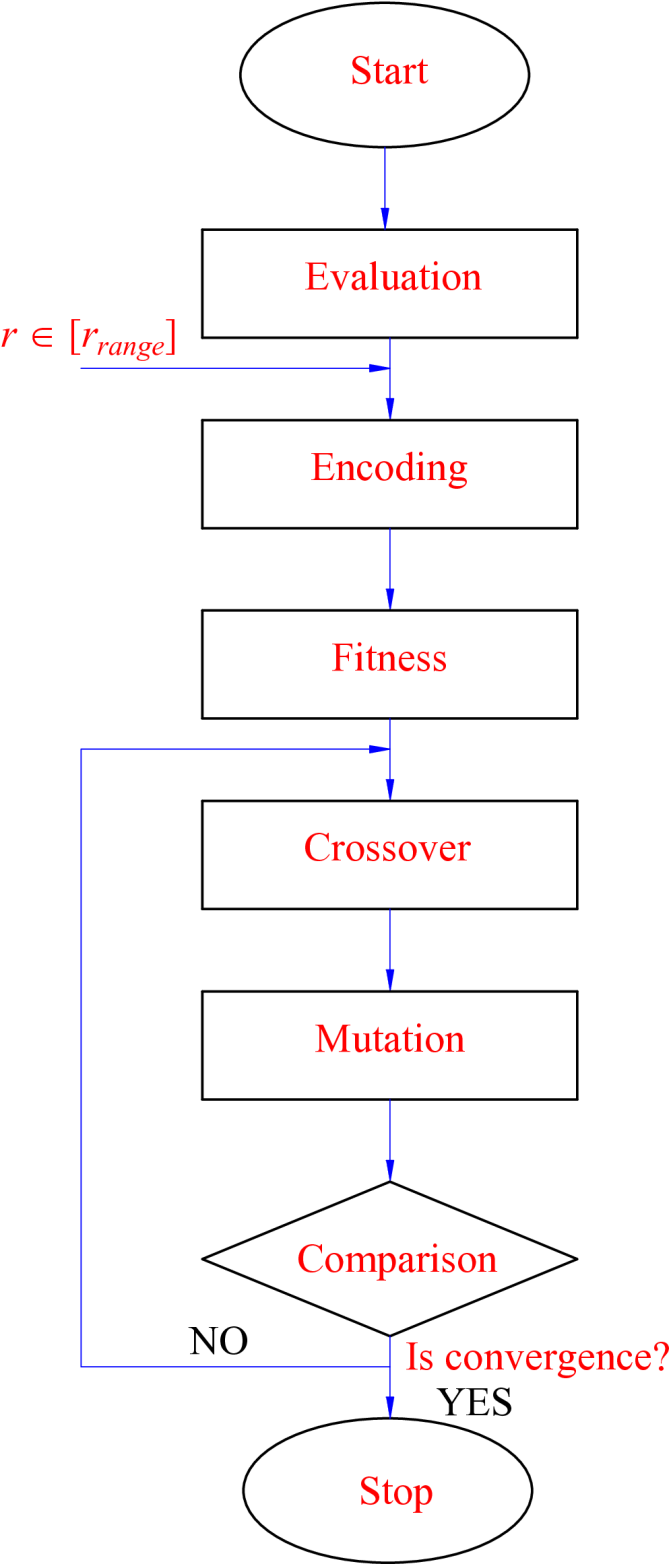
Genetic algorithm workflow.


The structure of GA is referred to in [61]. This computation process must go through six essential steps (Fig 5). Firstly, the effective range of the speed factor is verified to find *r*_*min*_ and *r*_*max*_. Then, encoding is performed in the second stage. This aims to change the problem’s goal from finding optimal parameters to finding the function’s fitness. A parameter vector (after being encoded) is illustrated in (42). Determining the fitness of function *J*(*φ*) means finding the dominant individuals in the population. The following work is done in the crossover and mutation stages to create unique individuals. Finally, the decoding and result evaluation process is performed to determine the convergence ability of the algorithm. The work will end when and only when the optimal value is found (satisfying the given condition) or the algorithm has run sufficient generations (44).

$$\varphi ={\left[\begin{array}{cccc} \varphi_{1} & \varphi_{2} & ... & \varphi_{n} \end{array}\right]}^{T}$$ (42)

$$f_{optimal}=fitness\left(J\left(\varphi \right)\right)$$ (43)

$$\mathrm{\backslash [}RMS\left(x_{1}\right)\overset{\min\;}{\to }r_{optimal}\mathrm{\backslash ]}$$ (44)

**Fig 6:**
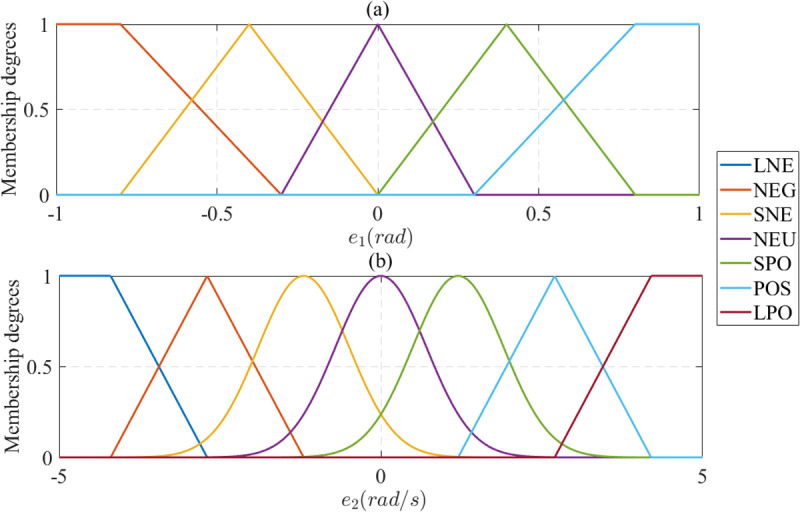
Membership functions. (a) *e*_1_ input; (b) *e*_2_ input.


In this work, the proportional parameter (*k*_*p*_) and the derivative parameter (*k*_*d*_) are tuned using a dynamic fuzzy algorithm instead of fixed selection. Combining ADRC with fuzzy logic control effectively improves the system adaptation under different operating conditions. This unique combination completely differs from the fixed parameter selection mentioned in previous ADRC studies. The structure of the proposed fuzzy controller consists of two inputs and one output. The errors *e*_1_ and *e*_2_ are considered the fuzzy controller’s first and second inputs, respectively. The first input (Fig 6a) is computed through five membership functions, described by (45) and (46). An expansion in the membership functions is seen in Fig 6b (the second input), where the number of membership functions is increased to seven. These functions are calculated by (47) and (48).

$$\mu_{F}\left(e_{1};a,b,c,d\right)=\max\left(\min\left(\frac{e_{1}-a}{b-a},1,\frac{d-e_{1}}{d-c}\right),0\right)$$ (45)

$$\mu_{F}\left(e_{1};a,b,c\right)=\max\left(\min\left(\frac{e_{1}-a}{b-a},\frac{c-e_{1}}{c-b}\right),0\right)$$ (46)

$$\mu_{F}\left(e_{2};a,b,c,d\right)=\max\left(\min\left(\frac{e_{2}-a}{b-a},1,\frac{d-e_{2}}{d-c}\right),0\right)$$ (47)

$$\mu_{F}\left(e_{2};\sigma ,c\right)=e^{-\frac{{\left(e_{2}-c\right)}^{2}}{2\sigma^{2}}}$$ (48)

The membership functions utilized in the fuzzy system, as defined in Equations 45 to (48), are constructed using triangular, trapezoidal, and Gaussian shapes. Triangular membership functions, known for their rapid response characteristics and computational efficiency, are employed for the input variable *e*_1_, which benefits from fast adaptation. In contrast, the input variable *e*_2_ exhibits high sensitivity, where overly steep transitions may lead to instability. Gaussian membership functions are adopted as a smoother alternative to the conventional triangular functions to mitigate this. Additionally, the saturation boundaries within the fuzzy inference mechanism are represented by trapezoidal membership functions, which provide a controlled limiting behavior.

**Fig 7:**
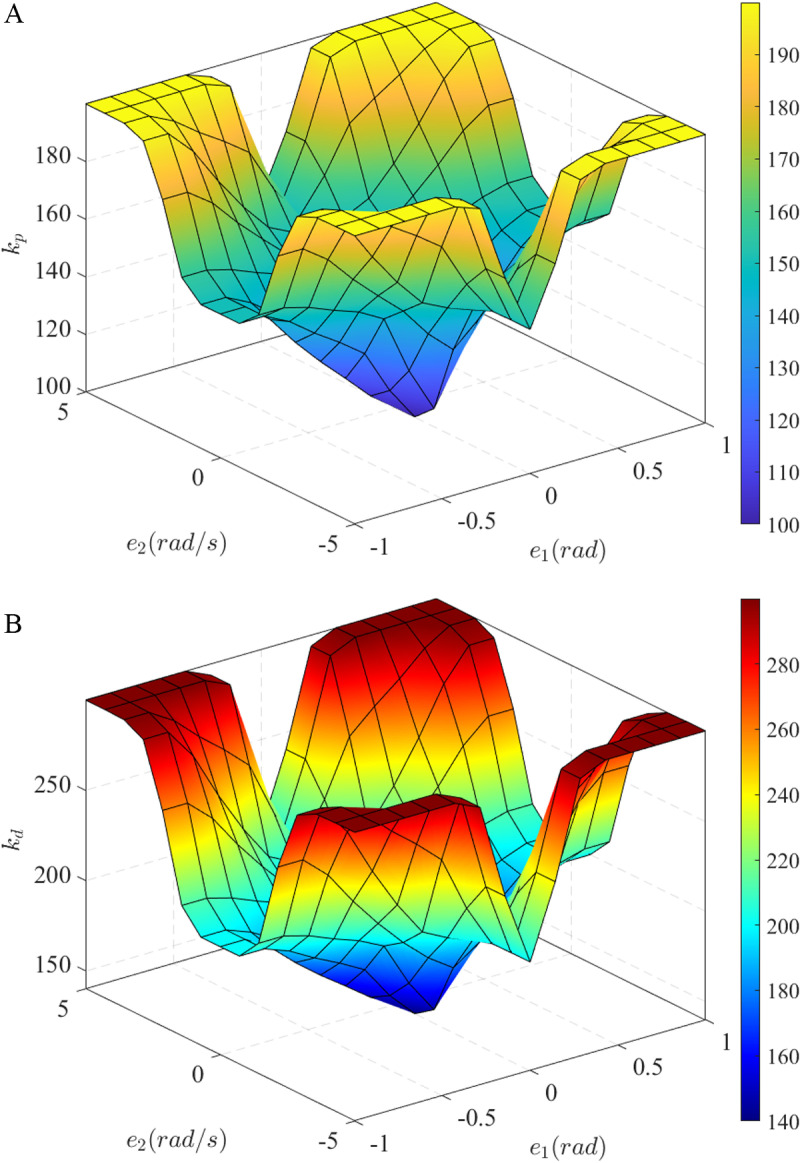
Fuzzy surfaces. (a) Fuzzy surface for *k*_*p*_; (b) Fuzzy surface for *k*_*d*_.

The relationship between the outputs and inputs of the fuzzy algorithm is illustrated in Fig 7a (*k*_*p*_) and Fig 7b (*k*_*d*_). This rule is listed in Table 3, where LNE is large negative, NEG is negative, SNE is small negative, NEU is neutral, SPO is small positive, POS is positive, and LPO is large positive. The fuzzy rules are empirically designed based on extensive prior simulations to ensure that the variations of the control parameters remain within predefined bounds. Moreover, the adaptability of these parameters is crucial, as they must respond appropriately to changes in the input error signals (*e*_1_ and *e*_2_). This flexibility is essential for maintaining both the stability and performance of the fuzzy control system under varying operating conditions.

**Table 3:** Fuzzy rules.

1^st^ input	2^nd^ input	Output	1^st^ input	2^nd^ input	Output
NEG	LNE	POS	NEU	SPO	NEU
NEG	NEG	POS	NEU	POS	SPO
NEG	SNE	SPO	NEU	LPO	SPO
NEG	NEU	SPO	SPO	LNE	POS
NEG	SPO	SPO	SPO	NEG	SPO
NEG	POS	POS	SPO	SNE	SPO
NEG	LPO	POS	SPO	NEU	NEU
SNE	LNE	POS	SPO	SPO	SPO
SNE	NEG	SPO	SPO	POS	SPO
SNE	SNE	SPO	SPO	LPO	POS
SNE	NEU	NEU	POS	LNE	POS
SNE	SPO	SPO	POS	NEG	POS
SNE	POS	SPO	POS	SNE	SPO
SNE	LPO	POS	POS	NEU	SPO
NEU	LNE	SPO	POS	SPO	SPO
NEU	NEG	SPO	POS	POS	POS
NEU	SNE	NEU	POS	LPO	POS
NEU	NEU	NEU			

## 3. Simulation and result

**Fig 8:**
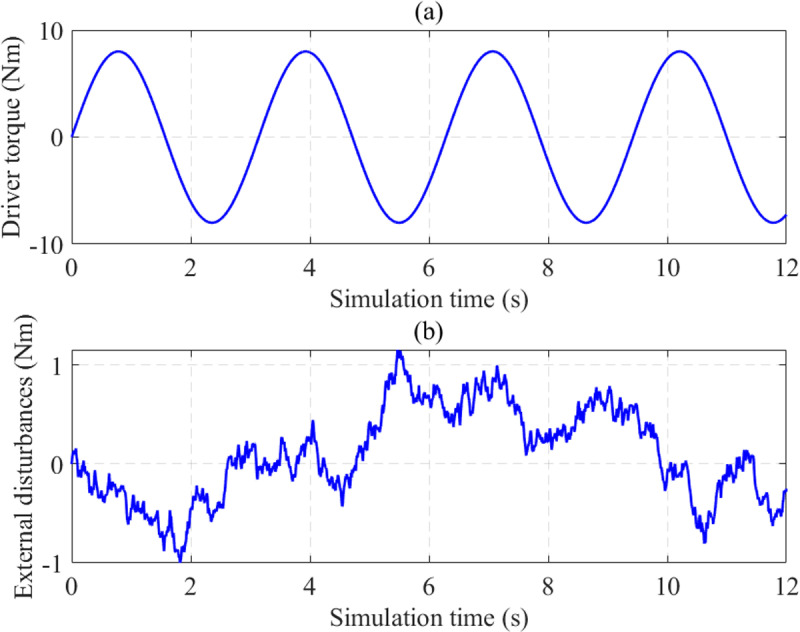
Simulation inputs. (a) Driver torque; (b) External disturbances.

Simulations are performed to investigate the performance of the proposed algorithm. The technical parameters of the EPS system and vehicle model are listed in Tables 1 and 2. Driver torque is shown in Fig 8a as the input signal. External disturbances depict environmental conditions’ influence (Fig 8b).

The simulation process is performed by a high-performance PC with an Intel Core i9-12900K CPU and 32GB of RAM. The variation of the output state variables is investigated in two cases, corresponding to two different speed values (*v*_1_ = 25 km/h and *v*_2_ = 70 km/h). The first describes the vehicle moving in congested conditions, while the second refers to the vehicle moving on a suburban road or highway. This aims to investigate the system response to changes in vehicle speed. The results obtained from the proposed algorithm are compared with other control methods, consisting of Proportional-Integral-Derivative (PID) and Linear Quadratic Tracking (LQT) Control. These two controllers are among the most commonly employed in linear system control owing to their structural simplicity and low implementation cost. As such, they serve as appropriate reference baselines for evaluating the performance of the proposed control strategy.

The parameters of PID and LQT controllers are calculated using a loop algorithm to optimize the control performance. For the proposed controller, the values of *k*_*p*_ and *k*_*d*_ are tuned by the dynamic fuzzy technique discussed in Section 2. The parameters of the tracking differentiator are determined by the genetic algorithm with the initial conditions referred to in [61]. The speed factor (*r*) value is 998.5; critical gain (*b*_0_) is 166.67. The coefficients for the conventional PID controller are *k*_*p*_ = 10.5, *k*_*i*_ = 9.2, and *k*_*d*_ = 0.2.

### 3.1. Low speed (v_1_)

**Fig 9:**
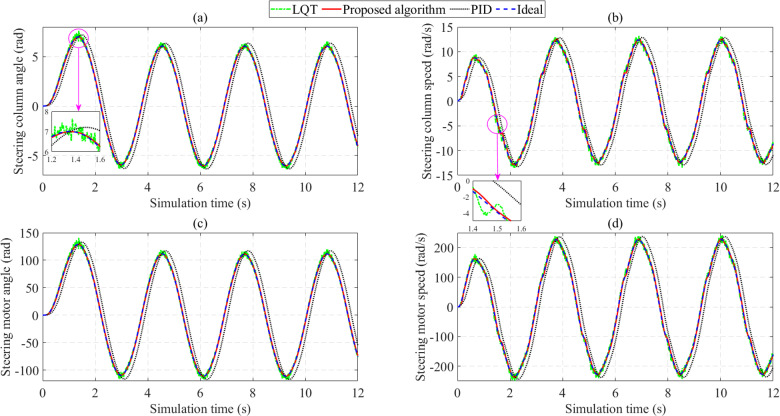
Change of EPS outputs (*v*_1_ = 25 km/h). (a) Steering column angle; (b) Steering column speed; (c) Steering motor angle; (d) Steering motor speed.

The variation of the state variables over time when the car moves at a speed of *v*_1_ = 25 km/h is illustrated by the subplots in Fig 9. Fig 9a provides information about the steering column angle, considered the controlled object in this article. Looking at this figure more closely, one can see that the output signals from the different controllers follow the ideal value. The output obtained from the PID controller is delayed compared to the other two controllers, leading to an increase in error. The simulation results show that the PID control’s maximum and RMS errors are 1.740 rad and 1.103 rad, respectively. Compared with the conventional PID, the LQT algorithm performs better in reducing system error. The calculation results show that the LQT algorithm’s maximum and RMS errors are only 0.625 rad and 0.134 rad, respectively. Although the LQT control signal closely follows the reference signal, the chattering phenomenon occurs strongly. This is due to the negative influence of sensor noise (the LQT control method necessitates obtaining all state variables signals). The proposed algorithm is capable of thoroughly solving the above problems. According to the article’s findings, the signal obtained from the proposed controller closely follows the reference signal with minor errors, only 0.136 rad for the peak value and 0.082 rad for the RMS value. The phase lag and chattering phenomena are completely eliminated once this technique is applied to control the EPS system.

The variation of steering column speed (*x*_2_) is illustrated in Fig 9b. Under the influence of external disturbances (Fig 8b), the output signals are no longer smooth curves. The most significant error belongs to the PID control, caused by the phase lag phenomenon. Under the influence of sensor noise, the LQT’s RMS error is up to 5.120%. A significant improvement is seen when the proposed technique is applied to control the automotive EPS system. According to the calculation results, the system’s maximum error is only 0.799 rad (about half that of LQT), while the RMS error is only 3.240%.

The steering motor angle variation (Fig 9c) is similar to the steering column angle. Although the value of the steering motor angle is much larger than that of the steering column angle, their RMS error rates are similar. Steering motor speed error is slightly reduced compared to steering column speed error (Fig 9d), except for PID control.

**Fig 10:**
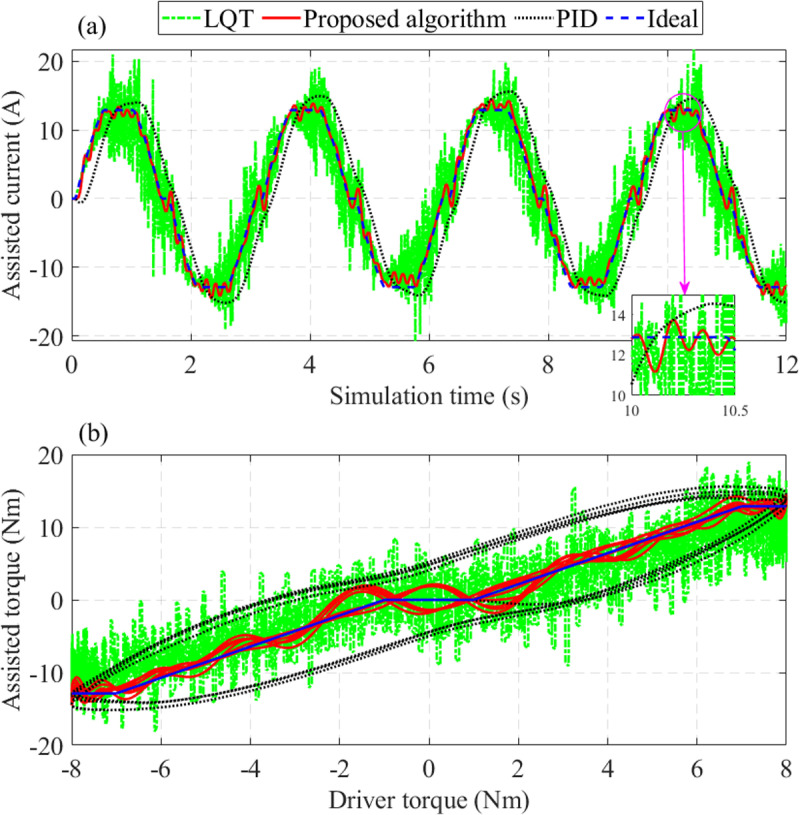
Change of assisted performance (*v*_1_ = 25 km/h). (a) Assisted current, (b) Relationship between assisted torque and driver torque.

Regarding the assisted current (*x*_5_), the output signal is heavily chattering once the system is controlled by the LQT technique (Fig 10a). The signal obtained by the PID controller is lagging in phase. In contrast, the results obtained by the proposed controller tend to follow the ideal signal with minor errors. The LQT controller’s maximum error is up to 15.929 A, about twice as large as the PID controller’s (7.307 A). The RMS error of the proposed algorithm is only 9.651%, which is much lower than that of the LQT (32.872%) and PID (43.395%).

The dependence of the assisted torque on the driver torque is illustrated in Fig 10b. Looking at this figure more closely, we can see that the error of the PID controller is enormous. The signal obtained by the LQT controller is negatively affected by the chattering phenomenon, which results from sensor noise. In contrast, the results obtained from the proposed controller follow the ideal signal with a small error. This error is because the actual system is affected by external disturbances while the ideal model is not.

**Fig 11:**
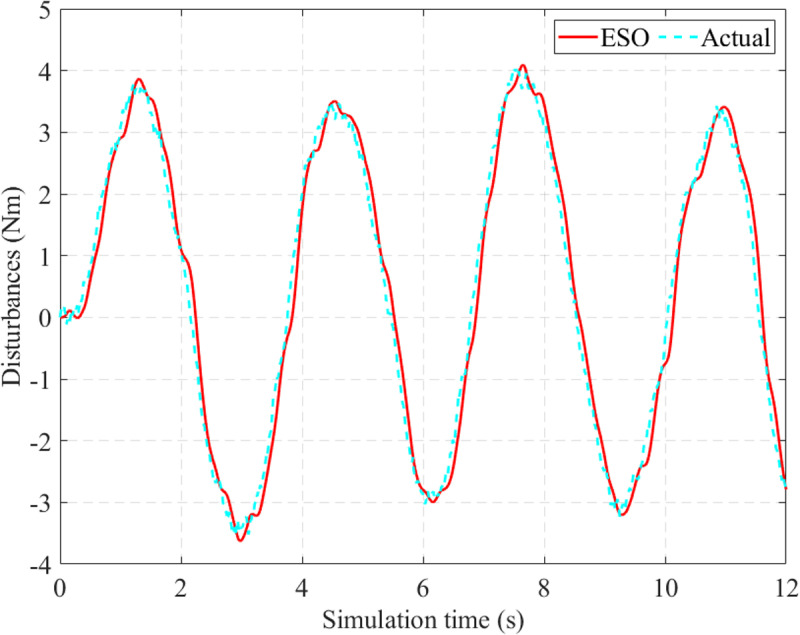
Observed disturbances (*v*_1_  = 25 km/h).

**Note:** The results mentioned in this article have been rounded after calculation.

In this work, disturbances are estimated by the ESO through the augmented variable (*x*_6_). The results in Fig 11 show that the observed signal tends to follow the actual signal with average errors (only 9.565% for RMS value and 1.669% for mean value).

### 3.2. Average speed (v_2_)

It is necessary to investigate the output results when the vehicle is moving at a higher speed to evaluate the performance of the proposed algorithm. In this article, we investigate the performance of the EPS system when the vehicle is steering at *v*_2_ = 70 km/h.

**Fig 12:**
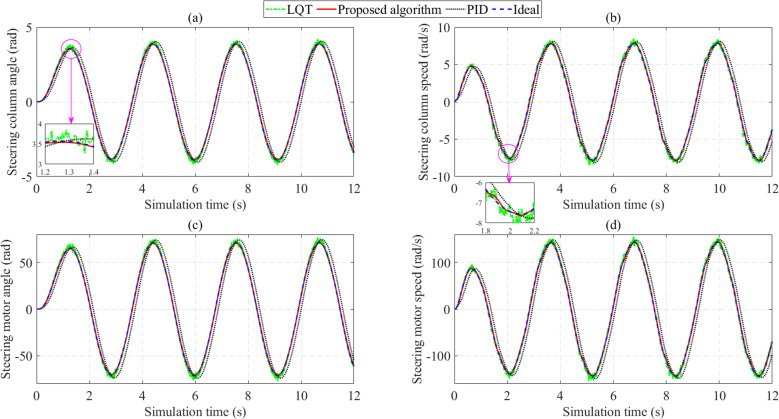
Change of EPS outputs (*v*_2_ = 70 km/h). (a) Steering column angle; (b) Steering column speed; (c) Steering motor angle; (d) Steering motor speed.

Fig 12 shows the changes in steering column angle, steering column speed, steering motor angle, and steering motor speed over time. The output values are generally reduced compared to the first case (*v*_1_ = 20 km/h). This is due to the degradation in power-assisted performance as the speed increases, consistent with the rule proposed in Fig 3. The simulation results reveal that the RMS errors of the steering column angle are 20.276% (PID), 3.319% (LQT), and 1.373% (Proposed algorithm), respectively. The PID control still has phase lag, which makes the system error worse. The signal from the LQT control, on the other hand, is heavily impacted by sensor noise (Fig 12a). The proposed controller has the slightest RMS error regarding steering column speed, whereas the LQT and PID controllers have many times more significant RMS errors (Fig 12b). The steering motor angle’s change trend is similar to the steering column angle’s (Fig 12c). This is also true for the steering motor and column speeds (Fig 12d). In general, the proposed algorithm’s RMS error is not more than 2% in this case, except for the steering column angle error (2.019%).

**Fig 13:**
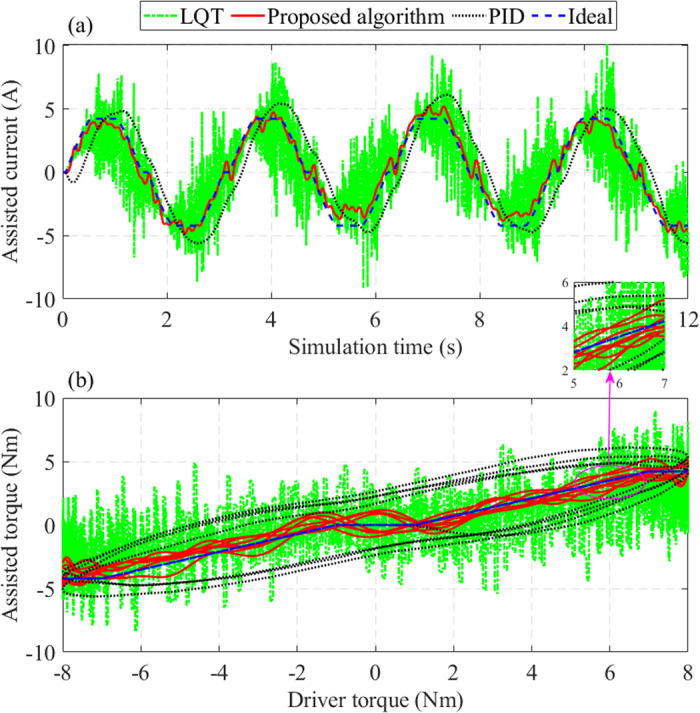
Change of assisted performance (*v*_2_ = 70 km/h). (a) Assisted current; (b) Relationship between assisted torque and driver torque.

The error of the assisted current in this condition is significant because of the reduction in the assisted torque (Fig 13). However, this is entirely consistent with reality. Reducing the assisted torque (or assisted current) improves safety when steering at high speed and avoids the rollover phenomenon. The results obtained by the proposed algorithm follow the ideal value with a much smaller error than the other two algorithms.

**Fig 14:**
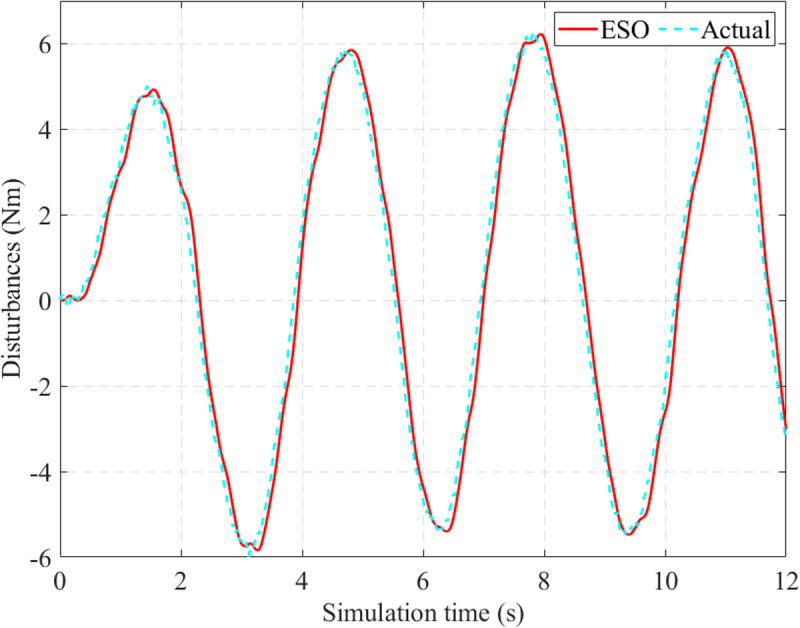
Observed disturbances (*v*_2_ = 70 km/h).

Although the power-assisted performance decreases with increasing speed, the observed disturbance error remains almost constant. In this case, the disturbance’s RMS error is 9.507%, 0.058% smaller than in the first case (Fig 14). The mean RMS error is 2.370%. It should be noted that the system disturbances include both internal and external disturbances. The random variation of external disturbances significantly impacts the observed error’s accuracy.

## 3.3. Discussion

The simulation results confirm that the proposed controller outperforms conventional controllers in tracking the reference signal. Specifically:

- The steady-state error is significantly smaller than with other controllers, like the PID-GA based on the MATLAB toolbox [5], traditional PI, ADRC techniques [10], and others [28,32].
- Unlike [10,14], the proposed controller almost eliminates the overshoot phenomenon, ensuring a more accurate and stable response.
- The adverse effects of sensor noise [18,20,21,23], caused by measuring physical signals, are lessened by the ESO’s use of state variables for estimation. This enhancement significantly improves system performance.
- The chattering phenomenon, which remains a persistent issue in robust control techniques like SMC and classical ADRC [26,27,29,33,62], is virtually eliminated in the investigated cases once the proposed solution controls the EPS system.
- Furthermore, the proposed controller shows lower power consumption than [7], validating its energy efficiency.
- Finally, the algorithm’s structure is much simpler than [35,36], which contributes to simplifying the computation process and increasing the opportunity for actual experimentation.

ADRC-based control can be highly effective in controlling automotive mechatronic systems to improve vehicle steering stability and ride comfort [63,64].

The proposed control mechanism demonstrates significant effectiveness in managing the EPS system. However, using linear models (including the linear ESO, linear TD, and linear state feedback control law) may lead to increased tracking errors and reduced accuracy in practical applications. Moreover, selecting membership functions and fuzzy rules based solely on prior simulation experience does not guarantee optimal system performance. Lastly, the proposed ESO may not provide sufficient effectiveness in the presence of heavy nonlinear disturbances. These limitations highlight the need for further investigation and improvement in future work.

## 4. Conclusion

The EPS system is capable of generating adaptive assisted torque that adjusts according to vehicle speed, thereby improving both steering feel and comfort. This article presents the novel control algorithm established based on the ADRC method, combined with soft computing techniques, to enhance the performance of the EPS system. The ESO is employed to estimate the output state variables, effectively mitigating the effects of sensor noise on system performance.

The algorithm’s performance is confirmed by the numerical simulation process. The article’s findings show that the output signals obtained from the proposed controller follow the ideal signal better than the conventional control methods. The steady-state error in the system state variables is inconsiderable, while the observed disturbance error remains within acceptable limits. Furthermore, the efficiency of the proposed method is maintained even in the presence of external random disturbances, thereby demonstrating its robustness and reliability.

Although the proposed algorithm demonstrates strong effectiveness in system control, several limitations must be addressed in the following works. Firstly, it has been seen that as vehicle speed increases, the error in the assisted current remains significant. Secondly, the disturbance error observed is relatively large and requires further reduction to improve accuracy. Thirdly, the fuzzy rules must be optimized to ensure the system’s adaptability in various scenarios. The first issue can be addressed by combining two robust control mechanisms: ADRC and SMC. A nonlinear ESO should replace the linear ESO to enhance the accuracy in estimating system disturbances, which is a solution to the second issue. Finally, the fuzzy rules can be improved by applying artificial intelligence algorithms, such as machine learning and neural networks. Additionally, empirical studies are essential to validate the practical efficacy of the proposed algorithm. Addressing these drawbacks is considered as the motivation for further work.

## Abbreviation:

ACO: ant colony optimization
LSEF: linear state error feedback
ADRC: active disturbance rejection control
MIMO: multiple inputs and multiple outputs
BPNN: back propagation neural network
NEG: negative
EHPS: electrohydraulic power steering
NEU: neutral
EPS: electric power steering
PAS: power-assisted steering
ESO: extended state observer
PID: proportional-integral-derivative
GA: genetic algorithm
POS: positive
HPS: hydraulic power steering
PSO: particle swarm optimization
LNE: large negative
RMS: root mean square
LPO: large positive
SISO: single input and single output
LPV: linear parameter-varying
SNE: small negative
LQE: linear quadratic estimation
SPO: small positive
LQG: linear quadratic gaussian
TD: tracking differentiator
LQR: linear quadratic regulator
